# A Rare Case of Primary Paraganglioma of the Facial Nerve Canal

**DOI:** 10.5334/jbsr.3957

**Published:** 2025-05-13

**Authors:** Simon Claeys, Ingeborg Dhooge, Veroniek Van Driessche

**Affiliations:** 1 Ghent University Hospital, Ghent, Belgium

**Keywords:** paraganglioma, facial canal, facial glomus, glomus tumor, facial nerve, ^68^Ga‑DOTATATE

## Abstract

We report the case of a 54‑year‑old woman with 3‑year‑history of right facial paralysis. Computed tomography (CT) and magnetic resonance imaging (MRI) findings were highly suggestive of a primary paraganglioma (PGL) of the facial nerve canal (FNC). ^68^Ga‑DOTATATE positron emission tomography (PET)/CT confirmed the neuroendocrine nature of the tumor.

*Teaching point:* The combined imaging findings of permeative bone destruction, rapid wash‑in and wash‑out perfusion, and ^68^Ga‑DOTATATE tracer uptake in a lesion arising from the mastoid segment of the FNC are diagnostic of a primary PGL of the FNC and may obviate the need for histological confirmation.

## Introduction

Head and neck PGLs are rare, making up 0.6% of all head and neck tumors. They most commonly arise in the carotid body (40–60%), jugular foramen and middle ear (30%), along the vagal nerve at the ganglion nodosum (10%), the sympathetic trunk (4%), and exceptionally in other locations in the head and neck area [1, 2]. Although secondary involvement of the FNC may occur with jugulotympanic PGLs, a primary PGL of the FNC is exceedingly rare with only 29 cases reported in literature. The clinical presentation is variable, but most patients present with facial nerve dysfunction and/or pulsatile tinnitus [1, 3]. Imaging plays an essential role in the diagnosis, with the most recent World Health Organization (WHO) classification of 2022 classifying all PGLs as malignant and placing the emphasis on functional imaging for the evaluation of disease extent [2].

## Case Report

A 54‑year‑old woman was referred to the neurology department for 3‑year history of right‑sided facial paralysis. In 2020, she experienced sudden right‑sided facial weakness, gradually progressing to near‑complete paralysis over the following months. Ten years prior, she was treated for breast cancer with complete remission at annual follow‑up. No other significant medical history was mentioned.

Physical examination revealed House–Brackmann (HB) V–VI right‑sided facial paralysis. Electromyography showed significant reduction of compound action potential in the right orbicularis oculi and oris muscles. MRI of the head was reported as normal, so she was diagnosed with Bell’s palsy and treated with corticosteroids, yet without resolving the paralysis.

Three years later, she presented to the plastic surgery department, expressing her wish for surgical correction of the facial paralysis.

**Figure 1:**
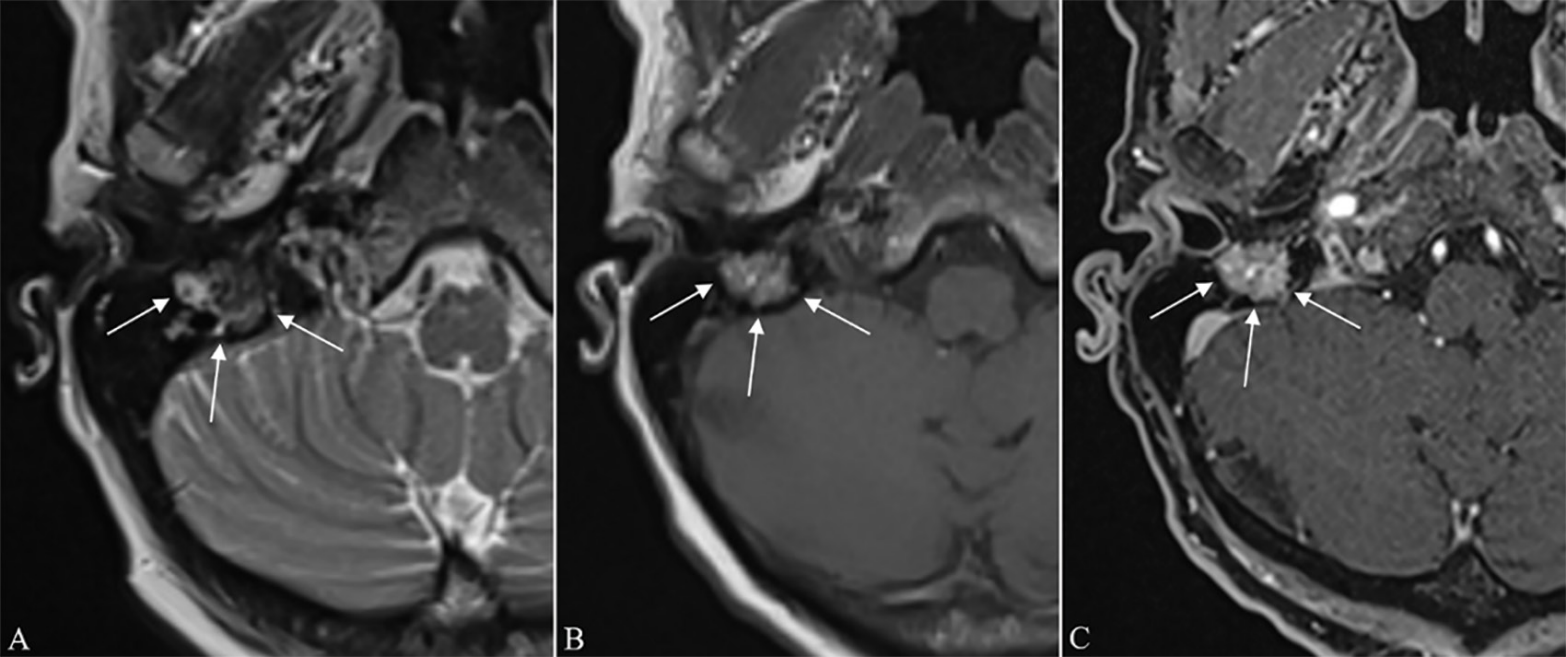
**(A)** Axial T2, **(B)** T1 before, and **(C)** fat‑saturated T1 after contrast administration. MR images show a mass (arrows) in the mastoid segment of the right FNC with heterogeneous T1/T2 signal, punctate hypointense foci suggesting flow voids, ‘salt‑and‑pepper’ appearance on T1, and avid contrast enhancement.

**Figure 2:**
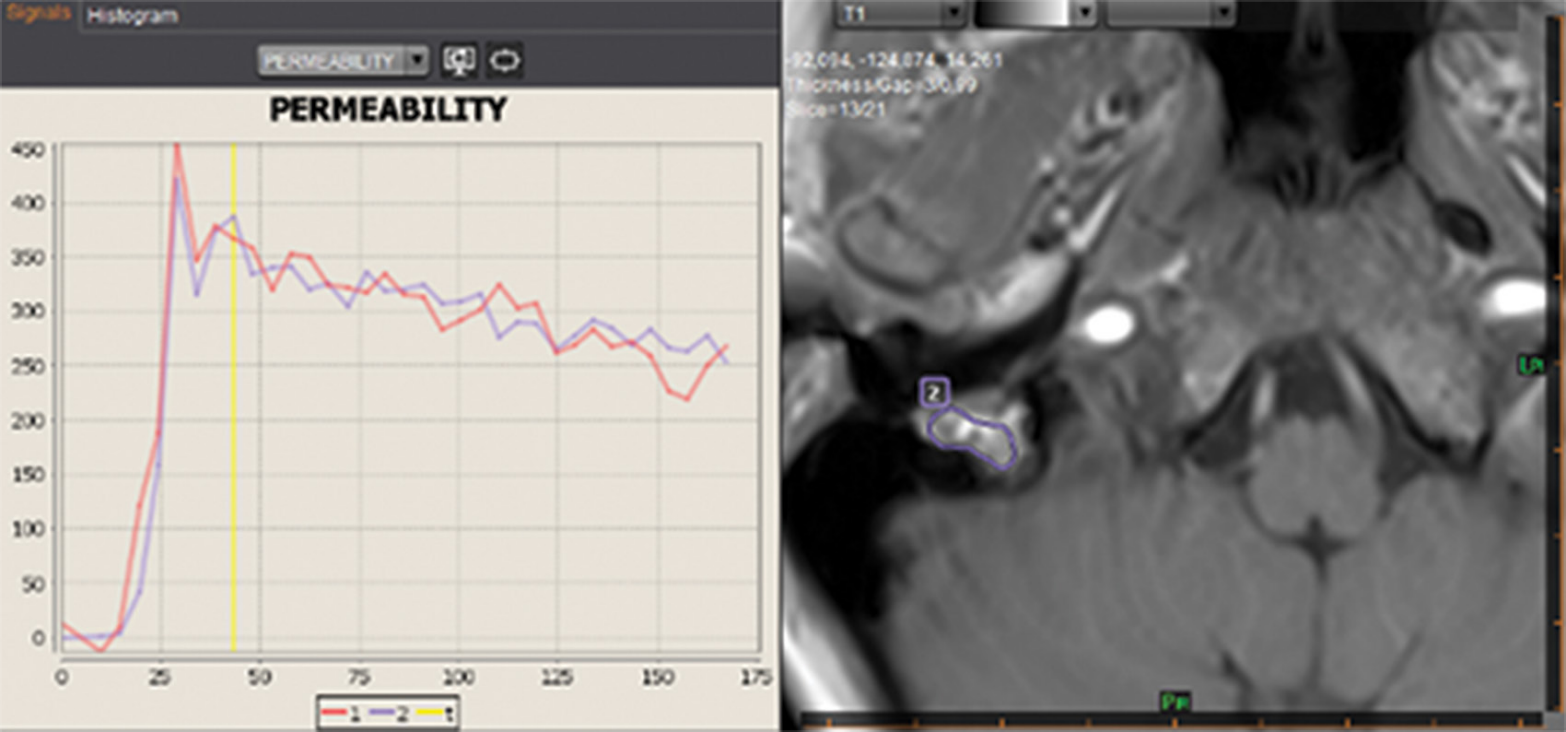
Axial T1‑weighted DCE perfusion MRI time–intensity curve demonstrating rapid and intense wash‑in and wash‑out (ROI2), similar to the arterial curve of the ICA (ROI1). Region of interest (ROI).

On otoscopy, a red, pulsating mass was seen protruding into the postero‑inferior part of the external auditory canal. MRI revealed a strongly enhancing mass in the right mastoid segment of the FNC and stylomastoid foramen (Figure 1). Retrospective analysis of the MRI performed 3 years earlier showed the mass, with only slight volume increase. Dynamic contrast‑enhanced (DCE) perfusion MRI showed a highly vascularized lesion with rapid and intense wash‑in and wash‑out (Figure 2).

**Figure 3:**
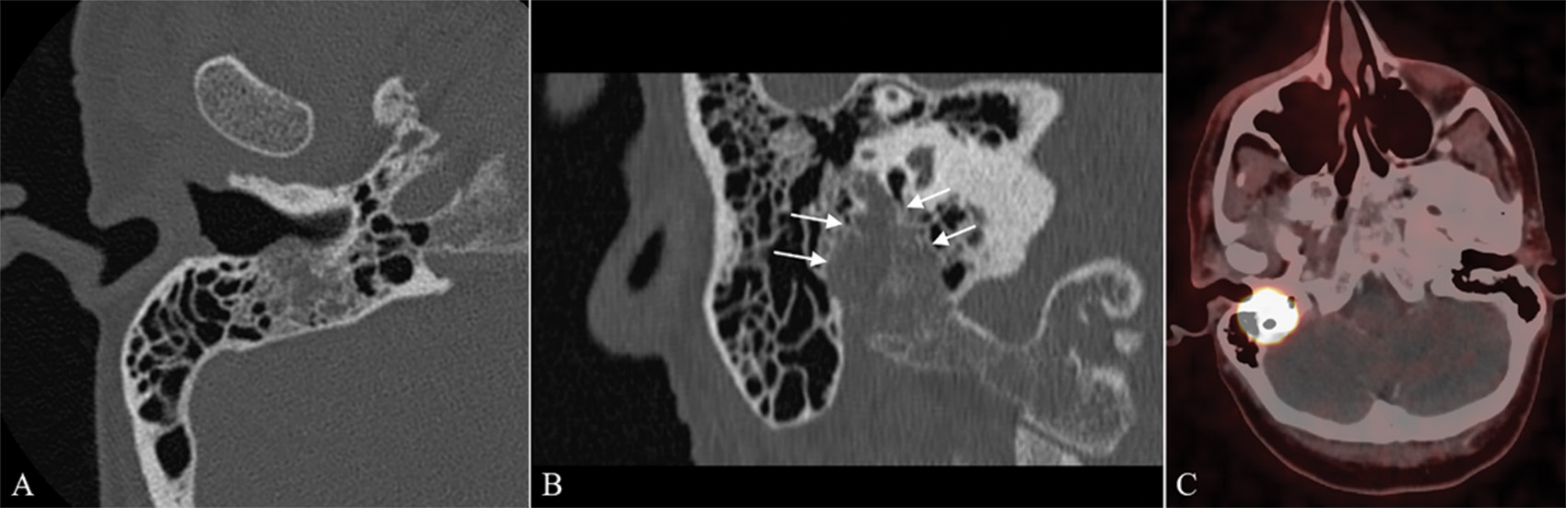
**(A)** Axial and **(B)** coronal CT scan at the level of the external auditory canal, demonstrating a soft tissue mass in the mastoid segment of the FNC and stylomastoid foramen, with permeative–destructive margins. **(C)** ^68^Ga‑DOTATATE PET/CT demonstrating intense tracer uptake.

Further evaluation by CT showed permeative bone destruction, with the lesion eroding into the external auditory canal but not involving the jugular foramen (Figure 3).

Differential diagnosis primarily consisted of facial nerve schwannoma and primary PGL. Given the concern for metastatic disease from previously treated breast cancer, ^18^F‑fluorodeoxyglucose (FDG) PET/CT was performed, which showed no lymph node involvement nor distant metastases. The lesion showed only mild FDG avidity.

To avoid a potentially harmful biopsy procedure, ^68^Ga‑DOTATATE PET/CT was performed, showing intense tracer uptake by the lesion, indicating somatostatine receptor expression (Figure 3), consistent with primary PGL of the FNC.

The patient was treated with stereotactic radiosurgery (SRS) (15 Gy at 90% isodose), with stable disease on follow‑up MRI.

## Discussion

Guild described the presence of paraganglia surrounding Arnold’s nerve, the ascending auricular branch of the vagus nerve, which enters the mastoid segment of the FNC just superior to the stylomastoid foramen [4]. This suggests that primary PGLs may arise in this location [5]. Most patients present with some degree of facial weakness, with acute onset and gradual worsening. Other symptoms include pulsatile tinnitus, facial spasms, hearing loss, otalgia, and aural fullness [3, 5].

On imaging, these tumors show similar characteristics as observed in PGLs elsewhere in the body, with avid contrast enhancement, flow voids with ‘salt‑and‑pepper’ appearance on T1WI, rapid wash‑in and wash‑out on DCE‑MRI, and permeative bone destruction on CT [5].

The differential diagnosis of a mass in the mastoid part of the FNC should include the most common facial nerve schwannoma, perineural spread from primary parotid or skin malignancies, and metastasis in an oncological setting. Venous–vascular malformations almost exclusively arise in the geniculate ganglion and tympanic segment [6].

Therapy consists of surgery and/or radiation therapy, with SRS being an increasingly applied, safe, and effective option in skull‑base PGLs [7]. Histological confirmation prior to therapy is challenging owing to the risk of bleeding and nerve damage associated with biopsy of these hypervascular tumors. ^68^Ga‑DOTATATE PET can confirm the neuroendocrine nature of the tumor noninvasively, with many studies proving its high sensitivity and specificity for PGLs [1]. Further, this study enables the exclusion of multifocal disease.

To our knowledge, this is the second published case of primary PGL of the FNC treated with SRS, and the second where ^68^Ga‑DOTATATE PET/CT was used in the workup.

## Conclusion

Head and neck PGLs can rarely occur primarily in the FNC. Multimodality imaging is essential for making the diagnosis, with ^68^Ga‑DOTATATE PET/CT being a valuable tool, especially when treatment with SRS is considered, as biopsy of these tumours harbors potential risks.
